# Glucagon-like Peptide-1 Receptor Agonists and Chronic Pain: Preclinical Antinociceptive Mechanisms, Indirect Metabolic–Functional Effects, and Clinical Considerations—A Narrative Review

**DOI:** 10.3390/jcm15155932

**Published:** 2026-07-29

**Authors:** Sung-woo Hyung, Ui Jin Park, Jeong Hwan Ryu, Siwook Chung

**Affiliations:** 1 Department of Anesthesiology and Pain Medicine, Eunpyeong St. Mary’s Hospital, College of Medicine, The Catholic University of Korea, Seoul 03312, Republic of Korea; sungwh312@nate.com (S.-w.H.); puj0902@gmail.com (U.J.P.); 2 Department of Anesthesiology and Pain Medicine, Uijeongbu St. Mary’s Hospital, College of Medicine, The Catholic University of Korea, Uijeongbu 11765, Republic of Korea; wjdghks7706@naver.com

**Keywords:** glucagon-like peptide-1 receptor agonist, chronic pain, pain management, pain-related outcomes, osteoarthritis, diabetic peripheral neuropathy, obesity, neuroinflammation, nociplastic pain, interventional pain

## Abstract

**Background/Objectives:** Glucagon-like peptide-1 receptor agonists (GLP-1RAs) are increasingly encountered in pain practice, but reported pain improvement may reflect direct antinociception, weight loss, metabolic change, or disease-specific effects. This structured narrative review examined these possibilities and relevant safety issues. **Methods:** PubMed/MEDLINE, Embase, and Web of Science Core Collection were searched through 15 May 2026. Peer-reviewed human studies, key mechanistic preclinical studies, systematic reviews and meta-analyses, and professional guidance relevant to pain outcomes or procedural safety were considered. Metabolic-only and non-peer-reviewed reports were excluded, and human pain-primary evidence was prioritized. **Results:** Preclinical data support plausible mechanisms involving spinal microglia, interleukin-10, beta-endorphin, neuroinflammation, and sensory-neuron signaling, but direct analgesic efficacy at systemic clinical doses has not been demonstrated. In STEP 9 (*n* = 407), mean WOMAC pain scores at 68 weeks changed from baseline by −41.7 points with semaglutide 2.4 mg and −27.5 points with a placebo; body weight changed by −13.7% and −3.2%, respectively. Liraglutide produced additional weight loss without superior knee-pain reduction. Diabetic peripheral neuropathy evidence was mainly structural or neurophysiologic; idiopathic intracranial hypertension and ROSE-010-treated irritable bowel syndrome showed disease-specific signals, whereas fibromyalgia- and opioid-related findings remained observational or hypothesis-generating. Gastrointestinal dysmotility, reduced intake, and lean-mass loss may offset functional benefits. **Conclusions:** GLP-1RAs are not established analgesics. Current human signals are best interpreted as indirect metabolic–functional or disease-specific effects. Future trials should use validated pain-primary outcomes, control for weight loss, and monitor treatment-related harms.

## 1. Introduction

Chronic pain is a multidimensional clinical condition shaped by nociceptive input, peripheral and central sensitization, immune signaling, metabolic status, sleep, mood, movement behavior, and social context [1]. In pain clinics, chronic pain commonly coexists with obesity, type 2 diabetes, insulin resistance, metabolic syndrome, obstructive sleep apnea, fatigue, and physical inactivity. These comorbidities can amplify pain through mechanical loading, adipose-tissue inflammation, peripheral nerve injury, altered cytokine signaling, sleep fragmentation, reduced physical capacity, and central amplification.

Glucagon-like peptide-1 receptor agonists (GLP-1RAs) have changed the treatment landscape for type 2 diabetes and obesity. Agents such as exenatide, liraglutide, dulaglutide, and semaglutide mimic or enhance GLP-1 receptor signaling and produce glucose-dependent insulin secretion, glucagon suppression, delayed gastric emptying, appetite reduction, weight loss, and broader cardiometabolic benefits [2,3,4,5,6]. As clinical exposure has expanded, pain physicians increasingly encounter patients receiving GLP-1RAs before consultations, rehabilitation, injections, radiofrequency procedures, and sedated interventions.

Within pain management, this topic is most relevant to multimodal analgesia, opioid minimization, non-opioid analgesic strategies, chronic pain phenotyping, interventional planning, procedural safety, and patient-centered outcomes. GLP-1RA exposure increasingly intersects with these domains because patients receiving these agents may present with obesity-associated osteoarthritis, diabetic neuropathy, headache disorders, visceral pain, opioid-related adverse effects, gastrointestinal intolerance, or planned sedated pain procedures.

Recent reviews have summarized preclinical and clinical evidence for GLP-1RAs across broad pain categories [2,3]. However, they have not primarily addressed the interpretive problem most relevant to pain practice: whether an observed improvement represents direct antinociception, indirect metabolic–functional benefit, disease-specific modification, contextual change, or a balance between benefit and treatment-related harm. This distinction is clinically important because pain improvement associated with weight loss or better function should not be interpreted as evidence of intrinsic analgesia, and structural or physiological improvement does not necessarily indicate symptomatic pain relief.

Accordingly, the overarching research question of this review is whether pain-related improvement observed during GLP-1RA therapy reflects direct antinociception or is better explained by indirect metabolic–functional and disease-specific effects. Our central working proposition is that most current human pain-related signals are more plausibly explained by indirect metabolic–functional or disease-specific mechanisms, whereas weight-independent direct analgesia remains unproven and treatment-related gastrointestinal, nutritional, and functional burdens may reduce the net clinical benefit. We therefore address four clinically relevant questions: which preclinical mechanisms support the biological plausibility of weight-independent antinociception; where human evidence is clinically meaningful; how direct, indirect, disease-specific, and contextual effects should be distinguished; and how treatment-related burdens may offset potential benefit.

## 2. Literature Search and Narrative Evidence Selection

### 2.1. Review Design

This article was designed as a structured narrative review integrating heterogeneous preclinical, human clinical, evidence-synthesis, and practice-guidance literature relevant to chronic pain management. The objective was not to answer a single PICO-based question or estimate a pooled treatment effect, but to interpret evidence across multiple pain phenotypes and distinguish preclinical antinociceptive mechanisms from indirect metabolic–functional, disease-specific, contextual, and treatment-related effects. Accordingly, the review was not conducted as a systematic review, scoping review, or meta-analysis, and full PRISMA reporting was not applied.

### 2.2. Information Sources and Search Strategy

PubMed/MEDLINE, Embase via Ovid, and Web of Science Core Collection were searched from database inception through 15 May 2026. Controlled vocabulary was used where incorporated into the database-specific strategy, together with free-text terms.

The exposure terms included “glucagon-like peptide-1 receptor agonist,” “GLP-1 receptor agonist,” semaglutide, liraglutide, exenatide, dulaglutide, tirzepatide, and ROSE-010. These were combined using Boolean operators with terms including pain, nociception, neuropathy, osteoarthritis, headache, migraine, idiopathic intracranial hypertension, visceral pain, irritable bowel syndrome, fibromyalgia, nociplastic pain, opioid-related outcomes, neuroinflammation, microglia, periprocedural management, gastric emptying, aspiration risk, rehabilitation, sarcopenia, and lean mass.

The strategy was adapted to the indexing system and syntax of each database. Reference lists and citation links of relevant reviews, clinical studies, mechanistic studies, and professional guidance documents were also examined to identify additional sources. Database-specific search blocks and syntax are provided in Appendix A Table A1.

### 2.3. Evidence Selection

Eligible sources included peer-reviewed randomized and nonrandomized human studies reporting pain, physical function, neurologic, visceral, opioid-related, or safety outcomes; mechanistic human studies; directly relevant systematic reviews and meta-analyses; preclinical studies evaluating GLP-1 signaling in pain-related neural, immune, peripheral nerve, or joint pathways; and professional society guidance relevant to sedation, delayed gastric emptying, aspiration risk, and interventional pain procedures.

Sources were excluded when they focused exclusively on glycemic control, body weight, cardiovascular, renal, or other general metabolic outcomes without clear relevance to pain mechanisms or pain practice. Conference abstracts, preprints, non-peer-reviewed reports, studies with unclear intervention identity, and substantially overlapping publications without additional relevant information were not used as primary evidence. Narrative reviews, editorials, and commentaries were used for contextual interpretation but were not treated as primary evidence of clinical efficacy.

Candidate sources were reviewed according to their relevance to the predefined clinical questions and the pain phenotypes covered in this review. The final synthesis comprised 44 sources. Because record-level screening counts and reasons for exclusion were not prospectively maintained as part of a systematic-review protocol, a retrospective PRISMA-style selection flow was not constructed.

### 2.4. Narrative Appraisal and Synthesis

The evidence base included randomized trials, observational studies, uncontrolled clinical studies, mechanistic and preclinical experiments, systematic reviews, and professional guidance. Because no single risk-of-bias instrument was applicable across all source types, formal cross-study risk-of-bias grading was not performed.

Instead, pivotal human studies were interpreted using predefined methodological considerations, including study design, sample size, comparator, whether pain was a primary or secondary outcome, use of validated pain or functional measures, follow-up duration, attrition, potential confounding by weight loss or metabolic improvement, and applicability to pain practice. Preclinical studies were used to establish biological plausibility and were not considered evidence of clinical analgesic efficacy. Professional guidance was used only to inform procedural and safety considerations.

Evidence was synthesized narratively by pain phenotype. Greater interpretive weight was given to controlled human studies with validated pain-primary outcomes, followed by studies reporting disease-specific structural or physiological outcomes. Observational, code-based, protocol, and preclinical findings were characterized as supportive, indirect, or hypothesis-generating, as appropriate. Quantitative synthesis and cross-phenotype certainty grading were not undertaken because of substantial heterogeneity in populations, interventions, comparators, outcome constructs, and study designs.

### 2.5. Terminology and Class Boundaries

In this review, GLP-1RA refers to selective glucagon-like peptide-1 receptor agonists unless otherwise specified. Tirzepatide and other dual or multi-agonists were included in the search to identify potentially relevant mechanistic, safety, or pain-management evidence but were not treated as interchangeable with selective GLP-1RAs. Findings from individual agents were not generalized to a class effect unless supported by sufficiently consistent evidence (Table 1).

## 3. Biological Rationale and Translational Limitations

The evidence discussed in this section is predominantly preclinical and should be interpreted as establishing biological plausibility rather than clinical analgesic efficacy. To date, there is no direct human evidence that systemically administered GLP-1RAs at clinically approved doses engage these spinal or sensory-neuron mechanisms sufficiently to produce weight-independent analgesia. Moreover, these findings do not establish direct modulation of cortical or subcortical networks involved in nociplastic pain.

### 3.1. GLP-1 Signaling Beyond Glycemic Control

Endogenous GLP-1 is an incretin peptide produced mainly by intestinal L cells and selected central neurons. It enhances glucose-dependent insulin secretion, suppresses glucagon, slows gastric emptying, reduces appetite, and contributes to postprandial metabolic regulation [4,5,6]. Pharmacologic GLP-1RAs were developed to overcome the short half-life of endogenous GLP-1 and differ substantially in molecular structure, half-life, dosing schedule, receptor exposure, central nervous system access, and weight-loss potency [4,7].

The relevance of GLP-1 signaling to pain arises from three overlapping domains. First, GLP-1 receptor activation may directly modulate nociceptive transmission and neuroimmune signaling. Second, GLP-1RAs modify metabolic and inflammatory environments that contribute to chronic pain. Third, changes in appetite, weight, sleep, activity, and comorbidity burden may reshape pain-related function and patient satisfaction.

### 3.2. Spinal Microglia, Interleukin-10, Beta-Endorphin, and Endogenous Opioid Signaling

The most specific preclinical analgesic mechanism involves spinal microglia. Activation of spinal GLP-1 receptors suppresses inflammatory and neuropathic pain hypersensitivity in animal models [8]. This effect appears partly mediated through microglial release of beta-endorphin, which can activate opioid receptors in the spinal dorsal horn [8,9,10]. Autocrine interleukin-10 signaling contributes to GLP-1 receptor-induced microglial beta-endorphin expression [9]. Non-peptide GLP-1 receptor agonism has also been reported to suppress inflammatory nociception through beta-endorphin release from spinal microglia [10].

This mechanism supports the biological plausibility of weight-independent antinociception. However, most experiments used animal models, intrathecal or experimental dosing, and mechanistic endpoints that do not automatically translate to systemic clinical dosing. They do not demonstrate clinically relevant target engagement after administration of currently marketed GLP-1RAs, nor do they establish modulation of supraspinal or cerebral pain networks in humans. Accordingly, the spinal microglia–interleukin-10–beta-endorphin pathway should be regarded as a preclinical mechanistic hypothesis rather than evidence of intrinsic analgesic efficacy in fibromyalgia or other nociplastic pain conditions.

**Figure 1:**
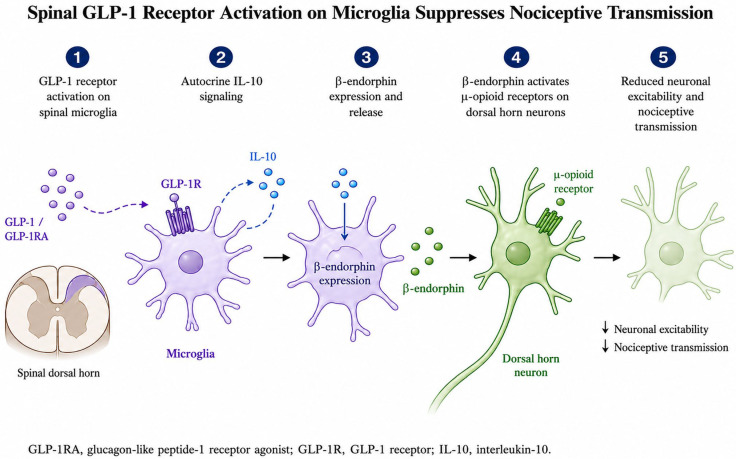
Proposed preclinical spinal microglial mechanism of GLP-1 receptor-mediated suppression of nociceptive transmission. Dashed arrows indicate proposed or indirect signaling relationships.

Figure 1 illustrates the proposed spinal microglial mechanism by which GLP-1 receptor activation may suppress nociceptive transmission.

### 3.3. Sensory-Neuron Mechanisms and TRPV1

Emerging experimental data suggest that GLP-1 and GLP-1-derived peptides may modulate peripheral sensory pathways. GLP-1-derived peptides have been reported to inhibit transient receptor potential vanilloid 1 (TRPV1)-related pain behavior without affecting thermoregulation [11]. This is mechanistically attractive because TRPV1 contributes to heat and inflammatory nociception. Whether marketed systemic GLP-1RAs engage this pathway at clinically relevant concentrations in human pain states remains unresolved.

Related experimental headache data also suggest that microglial GLP-1 receptor activation in the trigeminal nucleus caudalis can suppress migraine-like central sensitization in a recurrent nitroglycerin model [12].

These observations remain preclinical. In particular, suppression of migraine-like sensitization in an experimental trigeminal model should not be interpreted as evidence that systemic GLP-1RA therapy directly modifies human cortical pain processing, central sensitization, or nociplastic pain circuitry. Human mechanistic studies using quantitative sensory testing, functional neuroimaging, or other validated measures of central pain processing are currently lacking.

### 3.4. Neuroinflammation, Oxidative Stress, and Neuronal Injury

Neuroinflammation, oxidative stress, mitochondrial dysfunction, and microvascular injury contribute to chronic neuropathic and metabolic pain states. GLP-1RAs have been associated with anti-inflammatory and neuroprotective effects in preclinical and translational studies, including attenuation of oxidative stress, apoptosis, mitochondrial dysfunction, and inflammatory signaling [3,7]. These mechanisms are particularly relevant to diabetic peripheral neuropathy, where nerve injury reflects chronic metabolic and microvascular stress.

A critical distinction is required. Mechanisms that protect nerves or reduce neuroinflammation may eventually reduce pain, but they are not equivalent to acute symptomatic analgesia. Disease modification, neuroprotection, and analgesia should be measured separately in future clinical trials.

### 3.5. Systemic Metabolic and Functional Mechanisms

Indirect mechanisms are likely dominant in several clinical phenotypes. Weight loss reduces mechanical load on weight-bearing joints. Improved insulin resistance and glycemic control can reduce metabolic nerve injury. Reduced visceral adiposity may attenuate proinflammatory adipokine signaling. Better sleep apnea control, increased physical activity, and improved physical function may reduce pain interference and disability, without establishing direct modulation of central pain processing. These indirect effects are clinically meaningful but should not be mislabeled as direct analgesia.

**Figure 2:**
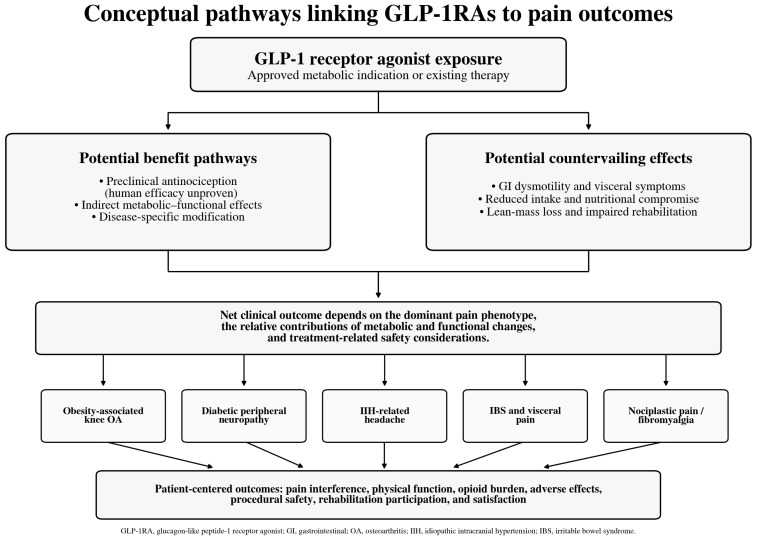
Conceptual pathways linking GLP-1 receptor agonists (GLP-1RAs) to pain outcomes. Potential metabolic, functional, disease-specific, and preclinical antinociceptive effects may be offset by gastrointestinal, nutritional, and rehabilitation-related adverse effects. The net clinical outcome depends on the pain phenotype and individual clinical context. GI, gastrointestinal; OA, osteoarthritis; IIH, idiopathic intracranial hypertension; IBS, irritable bowel syndrome.

Figure 2 summarizes potential benefit pathways and countervailing treatment-related effects through which GLP-1RA exposure may influence pain and function.

## 4. A Pain-Medicine Framework for Interpretation

A practical pain-medicine framework separates GLP-1RA-related evidence into preclinical direct antinociceptive mechanisms, indirect metabolic–functional pain improvement, disease-specific effects or modification, and contextual–functional effects. These potential benefit pathways must also be interpreted alongside countervailing treatment-related effects, including gastrointestinal dysmotility, visceral symptom exacerbation, nutritional compromise, loss of lean mass, and reduced rehabilitation participation. The net clinical outcome reflects the balance between these potentially beneficial and adverse pathways.

Preclinical direct antinociceptive mechanisms refer to GLP-1 receptor or GLP-1-derived signaling within nociceptive, spinal neuroimmune, sensory-neuron, or related experimental pathways that could theoretically reduce pain independently of weight loss or metabolic improvement. Spinal microglial, interleukin-10, beta-endorphin, and sensory-neuron data support this biological possibility [8,9,10,11]. However, direct analgesic efficacy at systemic clinical doses has not been demonstrated in humans, and no clinical evidence currently establishes direct modulation of cerebral nociplastic pain networks.

Indirect metabolic–functional pain improvement refers to pain improvement caused by weight loss, improved insulin resistance, reduced adipose inflammation, decreased mechanical loading, improved sleep, or improved physical capacity. This is likely relevant to obesity-associated knee osteoarthritis and may contribute to selected disease-specific or diabetic neuropathy phenotypes. In nociplastic pain, an indirect metabolic–functional contribution remains a research hypothesis rather than an established clinical effect.

Disease-specific effects or modification include alterations in disease physiology or the underlying pain-generating process. In diabetic peripheral neuropathy, this could include nerve structural or electrophysiological improvement. In osteoarthritis, it could include reduced synovial inflammation, reduced cartilage catabolism, or reduced progression to surgery, although definitive evidence remains incomplete.

Contextual–functional effects include changes in eating behavior, physical activity, fatigue, mood, sleep, opioid use, rehabilitation participation, and healthcare engagement. These effects may be valuable for patients but are especially vulnerable to confounding in observational studies (Table 2).

## 5. Evidence Across Clinical Pain Phenotypes

### 5.1. Obesity-Associated Knee Osteoarthritis

Obesity-associated knee osteoarthritis currently provides the strongest human evidence for clinically meaningful pain improvement during GLP-1RA therapy. This phenotype is biologically plausible because knee osteoarthritis pain is driven by mechanical loading, synovial inflammation, subchondral bone changes, muscle weakness, metabolic inflammation, and central pain processing. Osteoarthritis guidelines emphasize exercise and weight loss as core treatments for overweight or obese patients with knee or hip osteoarthritis [13,14].

The strongest trial signal comes from STEP 9. In adults with obesity and knee osteoarthritis with at least moderate pain, once-weekly semaglutide 2.4 mg plus lifestyle counseling produced greater reductions in body weight and WOMAC pain than placebo plus lifestyle counseling over 68 weeks; physical function also improved [15]. These findings make semaglutide 2.4 mg the most clinically relevant GLP-1RA for obesity-associated osteoarthritis pain at present.

However, interpretation is complex. The trial involved substantial weight loss, so pain reduction cannot be assumed to reflect direct analgesia. Mechanical unloading, reduced systemic inflammation, improved activity, and better function may all have contributed. Moreover, liraglutide data are less supportive. In a randomized trial of liraglutide after diet-induced weight loss in overweight or obese patients with knee osteoarthritis, liraglutide induced additional weight loss but did not reduce knee pain compared with placebo [16]. Therefore, weight loss does not uniformly translate into pain relief, and GLP-1RA effects may differ by agent, dose, population, run-in design, pain phenotype, and magnitude of weight loss.

Preclinical and translational osteoarthritis data suggest possible anti-inflammatory and anti-catabolic effects of GLP-1 receptor activation within joint tissues [17]. A recent rheumatology review concluded that knee OA is the arthritis phenotype with the clearest clinical signal, while effects on OA incidence and progression remain incompletely understood [29]. A population-based cohort study in patients with type 2 diabetes reported that GLP-1RA use, primarily dulaglutide, was not associated with lower osteoarthritis risk compared with dipeptidyl peptidase-4 inhibitor use [30]. These discordant signals reinforce the need for comparator-controlled and phenotype-specific trials.

For pain physicians, the defensible conclusion is that GLP-1RAs, particularly semaglutide 2.4 mg, may be clinically relevant in patients with knee osteoarthritis and obesity when used for approved metabolic indications. They should be framed as metabolic–functional therapies with possible additional anti-inflammatory or joint-specific effects, not as stand-alone analgesics.

Clinical take-home: Semaglutide-supported pain improvement in obesity-associated knee osteoarthritis is clinically relevant, but should be interpreted primarily as a metabolic–functional signal unless future trials demonstrate weight-loss-independent analgesia.

### 5.2. Diabetic Peripheral Neuropathy and Neuropathic Pain

Diabetic peripheral neuropathy is a biologically plausible target because it is linked to hyperglycemia, glycemic variability, insulin resistance, microvascular dysfunction, mitochondrial impairment, oxidative stress, and neuroinflammation. GLP-1RAs may affect this condition through glycemic improvement, weight loss, vascular effects, anti-inflammatory pathways, and direct neuroprotective mechanisms [18,31,32].

Clinical evidence is promising but does not yet support GLP-1RAs as symptomatic analgesics for painful diabetic peripheral neuropathy. In a small clinical study, GLP-1RA therapy was associated with improvement in neuropathy outcomes, mainly structural and morphological nerve measures supported by electrophysiological and clinical endpoints [18]. A prospective neurophysiology study reported improved clinical neuropathy scores and axonal excitability changes consistent with improved Na+/K+-ATPase pump function [33]. A meta-analysis suggested potential benefits of GLP-1RAs on peripheral nerve conduction velocity, but available studies remain limited by sample size, heterogeneity, and pain-specific endpoints [34].

Endpoint selection is central. Many diabetic neuropathy studies evaluate nerve structure, nerve conduction, corneal confocal microscopy, or neuropathy scores rather than pain intensity, allodynia, burning pain, sleep interference, or analgesic use. Nerve repair and pain relief are not identical. A substudy of the Qatar Study also highlights the complexity of interpreting incretin-based or metabolic therapies in neuropathy because active comparators, glycemic control, and neuropathy measures may influence conclusions [35].

Current evidence supports a potential disease-modifying or neuroprotective hypothesis for diabetic neuropathy, but not established symptomatic treatment efficacy. This cautious interpretation is consistent with a recent pain-medicine editorial that framed GLP-1RAs for neuropathic pain as a promising but still unproven therapeutic direction [36]. Future trials should separate painful from painless neuropathy and include validated pain outcomes, QST, nerve conduction studies, corneal confocal microscopy, intraepidermal nerve fiber density, glycemic variability, and concomitant analgesic use.

Clinical take-home: The diabetic peripheral neuropathy evidence supports a neuroprotective or disease-modifying hypothesis; it does not yet justify using GLP-1RAs as replacements for established neuropathic pain medications.

### 5.3. Headache, Migraine, and Idiopathic Intracranial Hypertension

Headache disorders provide a distinct line of evidence. The strongest clinical signal is in idiopathic intracranial hypertension (IIH), a disorder strongly associated with obesity, elevated intracranial pressure, visual risk, and disabling headache. In a randomized, placebo-controlled, double-blind trial, exenatide reduced intracranial pressure in women with active IIH at early and later time points, without a serious safety signal in the small study [19].

The mechanism is not conventional analgesia. GLP-1 receptor signaling may reduce cerebrospinal fluid secretion through effects on choroid plexus physiology, supported by preclinical work in raised intracranial pressure models [37]. Observational data suggest that GLP-1RA treatment in IIH may be associated with weight loss and favorable headache outcomes [38]. A large propensity-matched cohort study also reported associations between GLP-1RA therapy and lower medication use, reduced headache diagnoses, fewer visual signs, and fewer procedures over 1 year [39].

These findings are clinically important but disease-specific. They support further evaluation of GLP-1RAs as disease-specific interventions targeting intracranial-pressure physiology, not as general headache analgesics. Evidence for primary migraine remains less mature. Preclinical work suggests that microglial GLP-1 receptor activation in trigeminal pathways may modulate migraine-like central sensitization [12]. However, GLP-1RAs should not currently be regarded as migraine preventives outside approved metabolic indications or clinical trials.

Clinical take-home: Idiopathic intracranial hypertension is the clearest headache-related example of disease-specific GLP-1-responsive physiology; the available data should not be extrapolated to migraine prevention or nonspecific headache analgesia.

### 5.4. Visceral Pain and Irritable Bowel Syndrome

Visceral pain is another area in which GLP-1 pathway modulation has been evaluated in humans. ROSE-010, a short-acting GLP-1 analogue, has been studied as an on-demand treatment for acute abdominal pain attacks in irritable bowel syndrome (IBS). In a randomized, double-blind, placebo-controlled trial, ROSE-010 provided acute pain relief in a subset of patients with IBS [20]. A subsequent cross-analysis suggested dose- and time-dependent pain relief, with stronger signals in constipation-predominant and mixed IBS phenotypes [40]. A recent systematic review and meta-analysis also found that ROSE-010 reduced IBS pain compared with placebo, although nausea, vomiting, and headache occurred more frequently [41].

Conversely, long-acting GLP-1RAs used for diabetes or obesity commonly produce gastrointestinal adverse effects, including nausea, vomiting, constipation, diarrhea, and symptoms related to delayed gastric emptying [26]. In patients with pre-existing IBS, gastroparesis, chronic abdominal pain, or opioid-related bowel dysfunction, these effects could plausibly exacerbate bloating, abdominal discomfort, visceral pain, or pain interference, although direct prospective evidence in IBS populations remains limited. The net effect of GLP-1 pathway activation on visceral symptoms may therefore be bidirectional and may vary according to the specific agent, pharmacokinetic profile, dose-escalation phase, baseline gastrointestinal motility, IBS subtype, and concomitant medications.

Taken together, the ROSE-010 studies provide proof of principle that GLP-1 pathway modulation can influence acute human visceral pain. However, the short-acting, on-demand pharmacology of ROSE-010 differs substantially from that of long-acting GLP-1RAs used for metabolic indications. Accordingly, the acute pain-relief signal observed with ROSE-010 should not be extrapolated to a class-wide benefit of GLP-1RAs, and the current evidence is insufficient to support long-acting GLP-1RAs as treatments for chronic IBS-related pain.

Clinical take-home: ROSE-010 provides proof of principle for acute visceral pain modulation, whereas long-acting GLP-1RAs may exacerbate bloating, dysmotility, constipation, or abdominal discomfort in susceptible patients. The net effect is likely agent- and phenotype-dependent, and the available evidence does not establish a class-wide benefit for chronic IBS-related pain.

### 5.5. Fibromyalgia and Nociplastic Pain

Fibromyalgia and other nociplastic pain conditions remain unproven targets for GLP-1RA therapy. These conditions involve widespread pain, fatigue, sleep disturbance, cognitive symptoms, mood symptoms, autonomic complaints, and altered central pain processing. Although obesity, insulin resistance, sleep apnea, physical inactivity, and low-grade systemic inflammation may coexist in a subset of patients, such associations provide a rationale for investigating indirect metabolic–functional effects rather than evidence of direct modulation of nociplastic pain circuitry.

A large real-world propensity-matched cohort study using TriNetX data reported that GLP-1RA use in patients with fibromyalgia was associated with lower odds of opioid prescription, pain diagnostic codes, and fatigue diagnostic codes, while disability codes were not significantly different [21]. A 2026 review focused on fibromyalgia concluded that available human evidence remains limited to retrospective analyses and self-report, with no direct clinical trials establishing efficacy [22].

Importantly, no prospective human study identified in this review has demonstrated that systemic GLP-1RA therapy alters cortical or subcortical pain processing, central sensitization, temporal summation, conditioned pain modulation, or other validated markers of nociplastic pain. The available fibromyalgia evidence relies on diagnostic and prescription codes rather than validated pain, function, QST, or neuroimaging outcomes and is vulnerable to residual confounding related to body weight, metabolic status, sleep, fatigue, activity, prescribing behavior, and healthcare engagement. Accordingly, these findings support only a hypothesis that selected patients with concurrent metabolic dysfunction may exhibit indirect changes in pain-related function; they do not establish direct central analgesia.

Clinical take-home: Current fibromyalgia evidence is limited to observational associations and does not demonstrate direct modulation of cerebral or nociplastic pain circuits. GLP-1RAs should not be considered treatments for fibromyalgia outside approved metabolic indications or prospective clinical trials.

### 5.6. Opioid Exposure, Reward Pathways, and Opioid-Sparing Hypotheses

Chronic pain management often intersects with opioid exposure, opioid-induced constipation, medication-related fatigue, and reward-related eating behavior. GLP-1 receptor signaling has been investigated in reward and substance use pathways, and pain-management commentaries have proposed that GLP-1RAs may eventually influence opioid dependence or opioid-sparing strategies [23]. A randomized trial protocol is evaluating semaglutide in outpatients with opioid use disorder who continue to use nonprescribed opioids despite medications for opioid use disorder [24]. A large observational cohort of US veterans with type 2 diabetes suggested associations between GLP-1RA use and lower risks of developing several substance use disorders, but these data remain nonrandomized and not pain-specific [25].

Opioid-related GLP-1RA evidence should not be interpreted as analgesic evidence. Reduced opioid prescribing, craving, or substance-related outcomes may reflect reward modulation, healthcare engagement, metabolic improvement, prescribing patterns, or confounding. Prospective pain-specific studies are needed before GLP-1RAs can be considered opioid-sparing interventions in chronic pain.

Clinical take-home: Opioid and reward-pathway findings are relevant to pain-management research, but they should not be described as opioid-sparing analgesic evidence.

### 5.7. Other Neuropathic, Inflammatory, Cancer-Related, and Postsurgical Pain States

Preclinical evidence suggests that GLP-1 receptor activation can reduce inflammatory and neuropathic pain behaviors in several models [2,3,8,9,10,11]. These data justify further investigation in radiculopathy, chemotherapy-induced peripheral neuropathy, complex regional pain syndrome, cancer pain, and chronic postsurgical pain. Human evidence remains insufficient for clinical recommendations. At present, these conditions should be considered research domains.

Clinical take-home: Outside obesity-associated knee osteoarthritis, diabetic peripheral neuropathy, idiopathic intracranial hypertension, and ROSE-010-treated acute IBS pain attacks, clinical recommendations should be avoided until human pain-primary studies are available (Table 3).

## 6. Integrated Interpretation and Sources of Heterogeneity

Across phenotypes, the current human evidence does not support a uniform class-wide analgesic effect. The clearest pain-primary signal is in obesity-associated knee osteoarthritis, where semaglutide 2.4 mg produced substantial weight loss together with clinically meaningful improvements in WOMAC pain and function. The neutral liraglutide trial, despite additional weight loss, indicates that weight reduction alone is insufficient to explain response and suggests that agent, dose, achieved weight loss, treatment duration, run-in design, baseline pain severity, and concomitant lifestyle intervention may modify the clinical signal.

In diabetic peripheral neuropathy and idiopathic intracranial hypertension, the principal positive outcomes are structural or physiological rather than conventional analgesic endpoints. Improved nerve morphology or axonal function and reduced intracranial pressure may indicate disease-specific modification, but they should not be equated with symptomatic pain relief. In irritable bowel syndrome, ROSE-010 provides pain-primary proof of principle, but its short-acting, on-demand pharmacology limits extrapolation to long-acting metabolic GLP-1RAs. Fibromyalgia and opioid-related findings are based mainly on observational, code-based, or non-pain-specific outcomes and therefore remain particularly vulnerable to residual confounding.

Across studies, heterogeneity likely reflects differences in agent pharmacology, dose, treatment duration, baseline metabolic status, pain phenotype, magnitude and timing of weight loss, comparator and lifestyle cointerventions, and outcome selection. Studies using validated pain-primary endpoints and controlled comparators provide stronger evidence than studies relying on structural surrogates, diagnostic codes, or prescribing patterns. Direct analgesia remains unproven because existing human studies have not isolated pain effects from weight loss, metabolic improvement, disease-specific physiology, or contextual change.

## 7. Practical Implications for Pain Management

The main practical value of this topic for pain management is not immediate substitution of GLP-1RAs for analgesics. It is the need to recognize GLP-1RA exposure as part of multimodal pain care, opioid-sparing hypotheses, interventional planning, procedural safety, rehabilitation, and patient-centered outcomes.

### 7.1. Medication History and Indication

Pain physicians should increasingly expect patients to be receiving GLP-1RAs or GLP-1-based therapies for diabetes, obesity, cardiometabolic risk reduction, or other approved indications. A clinically useful medication history includes the exact agent, indication, dose, route, escalation phase, timing of last dose, duration of therapy, weight trajectory, gastrointestinal symptoms, diabetes regimen, renal function when relevant, and concurrent medications that affect bowel motility or sedation risk.

Current evidence does not support prescribing GLP-1RAs solely as analgesics in patients without approved indications. However, when an approved metabolic indication coexists with obesity-associated knee osteoarthritis, diabetic neuropathy, or IIH, pain and functional outcomes may reasonably be monitored as secondary, phenotype-specific treatment goals.

### 7.2. Gastrointestinal Adverse Effects and Potential Exacerbation of Visceral Symptoms

Nausea, vomiting, early satiety, delayed gastric emptying, diarrhea, and constipation are common GLP-1RA-related adverse effects [26]. These adverse effects may not only reduce treatment tolerability but may also worsen pain-related symptoms in selected patients; they may aggravate visceral discomfort or abdominal pain, particularly in patients with pre-existing IBS, chronic abdominal pain, autonomic neuropathy, gastroparesis, or opioid-induced bowel dysfunction.

The gastrointestinal effects of GLP-1RAs may overlap with those of opioids, anticholinergic agents, antidepressants, and other medications that alter gastrointestinal motility. In patients receiving chronic opioid therapy, GLP-1RA initiation or dose escalation should therefore prompt assessment of baseline bowel function, nausea, bloating, oral intake, hydration, and abdominal pain. New or worsening visceral symptoms should not automatically be attributed to the underlying pain disorder.

Clinicians should also recognize that the direction of the gastrointestinal effect may vary among patients. An intervention that improves symptoms in one visceral-pain phenotype may worsen constipation, bloating, or dysmotility in another. Dose escalation, pharmacokinetic profile, baseline motility, and concurrent medications should be considered when interpreting changes in abdominal pain.

### 7.3. Potential Countervailing Effects on Pain, Nutrition, and Rehabilitation

Potential pain-related benefits of GLP-1RA therapy may be offset by treatment-related gastrointestinal, nutritional, and functional burdens. Persistent nausea, vomiting, early satiety, or gastrointestinal discomfort can reduce energy and protein intake, impair hydration, disrupt sleep, increase fatigue and distress, and reduce adherence to medications or rehabilitation. These consequences may increase pain interference even when body weight or metabolic parameters improve.

Rapid or substantial weight loss may also include loss of lean mass. This is particularly relevant in older adults and in patients with frailty, sarcopenic obesity, advanced osteoarthritis, spinal stenosis, neurologic impairment, or chronic postsurgical deconditioning. Reduced muscle mass, weakness, and exercise intolerance may offset the benefits of mechanical unloading and limit participation in exercise-based rehabilitation, which remains a core component of chronic pain management.

Monitoring should therefore extend beyond body weight and glycemic control. Clinically relevant measures include the rate of weight loss, dietary and protein intake, hydration, muscle strength, gait and balance, physical activity, rehabilitation participation, visceral symptoms, pain interference, and functional trajectory. When adverse functional effects emerge, dose escalation, nutritional support, resistance exercise, and the overall treatment strategy should be reassessed in coordination with the prescribing clinician.

### 7.4. Interventional Pain Procedures, Sedation, and Gastric Emptying

GLP-1RAs slow gastric emptying and have raised concern about residual gastric contents during anesthesia or deep sedation. Periprocedural recommendations have evolved from broad medication-holding strategies toward individualized risk stratification [42,43]. For pain procedures, relevance depends on sedation depth, airway plan, patient positioning, aspiration risk, diabetes severity, obesity, obstructive sleep apnea, opioid use, gastrointestinal symptoms, and whether the patient is in a dose-escalation phase.

For procedures under local anesthesia without sedation, GLP-1RA therapy may have minimal practical impact. For procedures involving moderate sedation, deep sedation, prone positioning, limited airway access, or high aspiration risk, clinicians should follow current institutional and society guidance. High-risk features include active nausea or vomiting, abdominal distension, known gastroparesis, recent dose escalation, high-dose exposure, severe obesity, diabetes with autonomic neuropathy, and concurrent opioids. Gastric point-of-care ultrasound may assist aspiration-risk assessment in selected medically complex patients when local expertise is available [44].

### 7.5. Steroid Injections and Metabolic Context

Epidural, intra-articular, and peripheral corticosteroid injections can worsen hyperglycemia in patients with diabetes. GLP-1RA therapy may improve baseline glycemic control, but it should not be assumed to eliminate steroid-related hyperglycemic risk. Planning should consider baseline diabetes control, steroid type and dose, prior hyperglycemic response, glucose monitoring capacity, and coordination with the clinician managing diabetes. GLP-1RA therapy should not be viewed as a substitute for periprocedural glycemic planning.

### 7.6. Preserving Muscle and Bone Health During Weight Loss

Pain improvement during GLP-1RA therapy may depend on whether weight loss is accompanied by preservation of muscle function. Large weight reduction can include loss of lean mass, and this may be clinically relevant in older adults, patients with frailty, sarcopenic obesity, advanced osteoarthritis, spinal stenosis, or chronic postsurgical deconditioning [27]. Exercise may help preserve musculoskeletal health during GLP-1RA-associated weight reduction; a secondary analysis of a randomized trial found that exercise combined with GLP-1RA treatment had favorable implications for bone health compared with GLP-1RA treatment alone [28].

Pain physicians should integrate GLP-1RA-associated weight loss with resistance exercise, aerobic conditioning, gait training, nutrition, adequate protein intake, vitamin D and bone health assessment when appropriate, and functional goal-setting. The objective is not simply weight reduction, but improved pain-related function and satisfaction.

### 7.7. Patient Satisfaction and Shared Decision-Making

Patient satisfaction in pain medicine is influenced by pain relief, functional restoration, avoidance of adverse effects, perceived safety, procedural experience, and realistic expectations. GLP-1RAs may improve satisfaction if they reduce pain interference, facilitate rehabilitation, improve metabolic health, or reduce opioid burden. They may worsen satisfaction if nausea, constipation, cost, rapid weight loss, nutrition problems, or procedure scheduling issues are not anticipated. Shared decision-making should emphasize that GLP-1RAs are not established analgesics and that any pain-related relevance should be considered only in patients with an approved metabolic or disease-specific indication.

**Figure 3:**
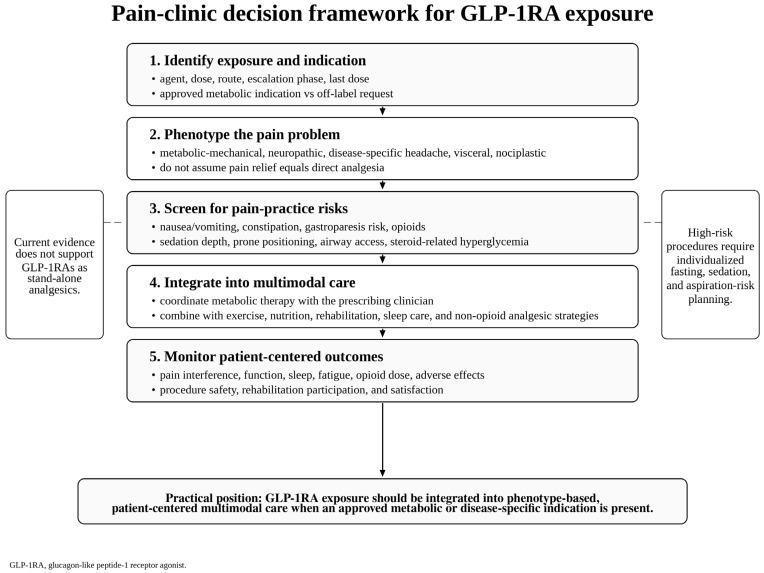
Pain-clinic decision framework for glucagon-like peptide-1 receptor agonist (GLP-1RA) exposure. The framework emphasizes medication identification, phenotype-based interpretation, screening for pain-practice and procedure-related risk, integration with multimodal pain care, and monitoring of patient-centered outcomes.

Figure 3 proposes a pain-clinic decision framework for patients already receiving or being considered for GLP-1RA therapy.

## 8. Future Directions and Trial Design

Future research should move beyond simple before-and-after pain scores. The key unanswered question is whether GLP-1RAs exert direct analgesic effects in humans and, if so, in which phenotypes and by what mechanism. Several design principles are essential.

First, trials should include weight-loss-adjusted analyses and, when feasible, active comparator groups that achieve similar weight reduction through non-GLP-1RA interventions. Without this, direct analgesia cannot be separated from mechanical and metabolic unloading. Second, studies should enroll patients according to pain phenotype, not diagnosis alone. Osteoarthritis trials should distinguish inflammatory, mechanical, neuropathic-like, and nociplastic features. Diabetic neuropathy trials should distinguish painful from painless neuropathy. Fibromyalgia trials should stratify by obesity, insulin resistance, sleep apnea, and inflammatory markers.

Third, trials should use multidimensional outcomes. Pain intensity is necessary but insufficient. Outcomes should include pain interference, physical function, sleep, fatigue, mood, opioid exposure, rescue analgesic use, QST, body composition, glycemic variability, inflammatory biomarkers, and disease-specific measures such as WOMAC, KOOS, FIQ-R, PROMIS domains, neuropathy scores, nerve conduction studies, corneal confocal microscopy, or intraepidermal nerve fiber density. Fourth, studies should include mediation analyses to estimate how much pain improvement is explained by weight loss, glycemic change, inflammatory markers, sleep improvement, or functional gains.

Finally, future studies should prespecify whether the target construct is symptomatic analgesia, disease modification, metabolic–functional improvement, or opioid-related behavioral change. Reports should include drug identity, dose, dose-escalation phase, discontinuation, absolute weight change, glycemic change, gastrointestinal adverse effects, concurrent lifestyle or rehabilitation interventions, and changes in analgesic regimens. Negative or neutral pain outcomes should be reported because failure to improve pain despite metabolic benefit may help identify phenotypes dominated by structural, neuropathic, nociplastic, or psychosocial mechanisms (Table 4).

## 9. Limitations of Current Evidence

Several limitations should temper interpretation. First, most evidence for direct analgesia comes from animal models, often using intrathecal or experimental dosing paradigms that do not mirror systemic clinical dosing. Second, human pain-primary trials remain limited and concentrated in a small number of phenotypes. Third, weight loss, glycemic improvement, lifestyle intervention, and increased clinical contact are major confounders. Fourth, observational studies often rely on diagnostic codes, prescription records, or procedure rates rather than validated patient-reported pain outcomes. Fifth, GLP-1RAs differ pharmacologically, and findings with semaglutide should not automatically be generalized to liraglutide, dulaglutide, exenatide, tirzepatide, or investigational multi-agonists. Sixth, chronic pain diagnoses are heterogeneous and may contain nociceptive, neuropathic, nociplastic, inflammatory, and psychosocial components in varying proportions. Seventh, because this article is a narrative review, selection and interpretation bias cannot be eliminated; the structured search improves transparency but does not replace a systematic review when quantitative effect estimates or formal certainty grading are required.

Furthermore, human mechanistic studies capable of testing direct central analgesia are lacking. No available study has combined GLP-1RA exposure with quantitative sensory testing, conditioned pain modulation, temporal summation, functional neuroimaging, or other validated biomarkers of nociplastic pain processing. Consequently, the current literature cannot determine whether the preclinical spinal and sensory-neuron mechanisms are engaged at systemic clinical doses in humans.

Because this narrative review was not conducted under a prospectively registered protocol, source selection and the relative emphasis assigned to individual studies depended partly on author judgment, and relevant evidence may have been missed. Formal design-specific risk-of-bias assessment and certainty grading were not performed; consequently, comparisons of evidentiary strength across phenotypes remain qualitative and may be affected by publication bias, selective outcome reporting, and residual confounding. The conclusions should therefore be interpreted as a clinically oriented synthesis rather than as definitive estimates of treatment efficacy.

These limitations do not undermine the relevance of GLP-1RAs to pain medicine. They define the research agenda. The field should move from broad claims about analgesia toward phenotype-specific, mechanism-informed, and comparator-controlled studies.

## 10. Conclusions

GLP-1RAs occupy an emerging intersection between metabolic medicine, neuroimmune modulation, and chronic pain care. Preclinical studies support putative antinociceptive mechanisms involving spinal microglia, interleukin-10, beta-endorphin, oxidative stress, neuroinflammation, and sensory-neuron pathways. Human evidence is most clinically developed in obesity-associated knee osteoarthritis, where semaglutide improves pain and function alongside substantial weight loss. In diabetic peripheral neuropathy, GLP-1RAs show preliminary nerve structural and functional signals, but symptomatic analgesic efficacy remains unproven. In IIH, exenatide provides a disease-specific signal through intracranial pressure reduction. In IBS, ROSE-010 demonstrates proof of principle for GLP-1 pathway modulation of acute visceral pain. In fibromyalgia and opioid-related outcomes, evidence remains observational and hypothesis-generating.

Potential benefits should be balanced against treatment-related burdens. Delayed gastric emptying, nausea, bowel dysfunction, visceral symptom exacerbation, reduced oral intake, nutritional compromise, and loss of lean mass may worsen pain-related function or interfere with exercise-based rehabilitation. These risks may be particularly relevant during dose escalation and in patients with IBS, gastroparesis, chronic opioid exposure, frailty, or sarcopenia. Accordingly, pain-related outcomes should be monitored bidirectionally rather than assuming that metabolic improvement will necessarily translate into pain relief or functional gain.

The most clinically useful conclusion is that GLP-1RAs are not established analgesics. Current human pain-related signals are more appropriately interpreted as indirect metabolic–functional or disease-specific effects, whereas direct neuroimmune and sensory-neuron mechanisms remain preclinical hypotheses. In particular, direct modulation of cerebral or nociplastic pain networks has not been demonstrated in humans. Pain physicians should consider GLP-1RA exposure within patient-centered multimodal care, including opioid adverse-effect overlap, gastrointestinal tolerability, sedation and aspiration-risk planning, steroid-related glycemic effects, rehabilitation, muscle preservation, and satisfaction. Clinically, the most relevant setting is a patient already receiving or eligible for GLP-1RA therapy for an approved metabolic indication who also has obesity-associated knee osteoarthritis, diabetic peripheral neuropathy, or idiopathic intracranial hypertension. GLP-1RAs should not be initiated solely for fibromyalgia, migraine, nonspecific chronic pain, or opioid-sparing purposes, and the ROSE-010 findings should not be extrapolated to long-acting GLP-1RAs.

Future research should avoid class-wide or indication-level overclaim and should distinguish symptomatic analgesia from metabolic benefit, disease modification, and contextual–functional effects using validated multidimensional outcomes. Further studies are needed to determine whether GLP-1-based therapies have a clinically meaningful role as phenotype-based adjuncts when used for approved metabolic or disease-specific indications.

## Figures and Tables

**Table 1:** Summary of Search Scope and Final Evidence Base.

Item	Description
Search timeframe	Database inception to 15 May 2026
Databases searched	PubMed/MEDLINE, Embase, and Web of Science Core Collection
Sources included in the narrative synthesis	44
Record-level identification and exclusion counts	Not prospectively maintained because the review was not conducted under a systematic-review protocol
Additional search information	Database-specific search strategies are provided in Table A1 in Appendix A.

**Table 2:** Mechanism-to-clinic interpretation framework.

Mechanism Category	Proposed Pathway	Best Supporting Evidence	Clinical Interpretation
Preclinical direct antinociceptive mechanisms	Spinal GLP-1 receptor activation, microglial interleukin-10, beta-endorphin release, opioid receptor activation, sensory-neuron modulation	Preclinical inflammatory and neuropathic pain models [8,9,10,11]	Biological plausibility only; clinically relevant target engagement and direct modulation of human nociplastic pain networks have not been demonstrated
Indirect metabolic–functional pain improvement	Weight loss, reduced mechanical joint load, improved insulin resistance, reduced adipose inflammation, sleep/activity improvement	Obesity-associated knee OA and metabolic pain phenotype data [13,14,15,16]	Clinically meaningful but should not be conflated with direct analgesia
Disease-specific effects or modification	Improved nerve morphology/function, reduced joint inflammation/catabolism, intracranial pressure reduction, visceral afferent or motility effects	DPN structural studies, OA preclinical data, IIH exenatide trial, ROSE-010 trials [17,18,19,20]	Promising for selected phenotypes; requires longitudinal validation
Contextual–functional effects	Changes in fatigue, activity, mood, eating behavior, opioid exposure, rehabilitation engagement, and healthcare contact	Fibromyalgia and opioid-related observational/protocol data [21,22,23,24,25]	Potentially important for patient satisfaction, but high risk of residual confounding
Countervailing treatment effects	Gastrointestinal dysmotility, nausea, vomiting, constipation or diarrhea, reduced oral intake, dehydration, lean-mass loss, fatigue, and reduced rehabilitation participation	GLP-1RA safety and body-composition/rehabilitation studies [26,27,28]	May worsen visceral symptoms or offset functional benefits, particularly during dose escalation and in frail or gastrointestinally vulnerable patients

**Table 3:** Evidence map for GLP-1RA-related pain outcomes.

Pain Phenotype	Best Current Evidence	Likely Dominant Mechanism	Clinical Interpretation
Obesity-associated knee OA	Semaglutide STEP 9 RCT positive for pain/function; liraglutide RCT negative for pain despite additional weight loss [15,16]	Weight loss, mechanical unloading, inflammation reduction, possible joint effects	Most clinically relevant phenotype; does not prove direct analgesia
Diabetic peripheral neuropathy	Small structural/neurophysiology studies and meta-analysis of nerve conduction [18,33,34]	Neuroprotection, metabolic improvement, oxidative stress reduction	Disease-modifying hypothesis; symptomatic analgesia unproven
IIH-related headache	Exenatide RCT reduced intracranial pressure; observational cohorts suggest favorable headache and procedural outcomes [19,38,39]	Reduced cerebrospinal fluid secretion, intracranial pressure reduction, weight loss	Disease-specific signal; not general headache indication
IBS/visceral pain	ROSE-010 randomized and cross-analysis data; meta-analysis positive for acute pain relief [20,40,41]	Visceral afferent and motility modulation	Proof of principle; long-acting GLP-1RAs may worsen gastrointestinal symptoms in susceptible patients; no class-wide benefit has been established.
Fibromyalgia/nociplastic pain	Propensity-matched real-world cohort and narrative synthesis; no direct RCTs [21,22]	Possible indirect changes related to weight, sleep, fatigue, activity, or metabolic inflammation; substantial residual confounding. Direct nociplastic mechanism unproven.	Hypothesis-generating only; no evidence of direct modulation of human cerebral pain circuits or established efficacy in fibromyalgia.
Opioid-related outcomes	Commentaries, observational SUD studies, and OUD trial protocols [23,24,25]	Reward modulation, behavior, prescribing patterns, metabolic improvement	Research domain; not evidence of analgesia or opioid sparing
CRPS, radiculopathy, cancer pain, postsurgical pain	Mainly preclinical or indirect evidence [2,3]	Neuroimmune modulation possible	No clinical recommendation at present

**Table 4:** Trial design recommendations for future pain studies of GLP-1RAs.

Design Domain	Recommended Approach	Rationale
Population	Enroll by phenotype, not diagnosis alone	OA, DPN, fibromyalgia, and headache are mechanistically heterogeneous
Comparator	Use placebo plus lifestyle, active weight-loss comparator, or metabolic comparator when feasible	Required to separate direct analgesia from weight-loss and metabolic effects
Primary endpoint	Use condition-specific validated pain outcomes	Examples include WOMAC pain for knee OA, neuropathic pain scales for painful DPN, and FIQ-R/PROMIS for fibromyalgia
Secondary endpoints	Assess pain interference, function, sleep, fatigue, mood, opioid dose, and rescue analgesic use	Captures patient-centered outcomes relevant to pain management
Mechanistic endpoints	Measure body weight, body composition, HbA1c, glycemic variability, hsCRP, adipokines, QST, nerve conduction, and imaging or tissue biomarkers	Enables mediation analysis and responder phenotyping
Safety endpoints	Track GI intolerance, constipation, nutritional compromise, discontinuation, sedation/procedure changes, and aspiration-risk events	Particularly relevant to sedated and interventional pain practice
Analysis	Perform mediation and subgroup analyses adjusted for weight loss, glycemic improvement, sleep, activity, and opioid exposure	Determines whether the clinical signal is direct, indirect, or contextual
Interpretation objective	Prespecify whether the hypothesis is direct analgesia, metabolic–functional benefit, disease modification, or opioid/contextual change	Prevents overclaim and makes neutral or discordant results clinically interpretable

## Data Availability

No new data were created or analyzed in this study. Data sharing is not applicable to this article.

## Abbreviations

The following abbreviations are used in this manuscript:

CRPS	Complex regional pain syndrome
DPN	Diabetic peripheral neuropathy
FIQ-R	Fibromyalgia Impact Questionnaire-Revised
GI	Gastrointestinal
GLP-1	Glucagon-like peptide-1
GLP-1RA	Glucagon-like peptide-1 receptor agonist
HbA1c	Glycated hemoglobin
IBS	Irritable bowel syndrome
IIH	Idiopathic intracranial hypertension
KOOS	Knee Injury and Osteoarthritis Outcome Score
OA	Osteoarthritis
OUD	Opioid use disorder
POCUS	Point-of-care ultrasound
PROMIS	Patient-Reported Outcomes Measurement Information System
QST	Quantitative sensory testing
RCT	Randomized controlled trial
SUD	Substance use disorder
TRPV1	Transient receptor potential vanilloid 1
WOMAC	Western Ontario and McMaster Universities Osteoarthritis Index

## Appendix A

**Table A1:** Database-specific literature search strategies.

Database/Platform	Search Period	Core Boolean Search Strategy
PubMed/MEDLINE	Database inception to 15 May 2026	(“glucagon-like peptide-1 receptor agonist”[Title/Abstract] OR “glucagon-like peptide-1 receptor agonists”[Title/Abstract] OR “GLP-1 receptor agonist”[Title/Abstract] OR “GLP-1 receptor agonists”[Title/Abstract] OR semaglutide[Title/Abstract] OR liraglutide[Title/Abstract] OR exenatide[Title/Abstract] OR dulaglutide[Title/Abstract] OR tirzepatide[Title/Abstract] OR “ROSE-010”[Title/Abstract]) AND (pain[Title/Abstract] OR nocicep*[Title/Abstract] OR neuropath*[Title/Abstract] OR osteoarthriti*[Title/Abstract] OR headache*[Title/Abstract] OR migraine*[Title/Abstract] OR “idiopathic intracranial hypertension”[Title/Abstract] OR “visceral pain”[Title/Abstract] OR “irritable bowel syndrome”[Title/Abstract] OR fibromyalgia[Title/Abstract] OR opioid*[Title/Abstract])
Embase via Ovid	Database inception to 15 May 2026	(exp glucagon like peptide 1 receptor agonist/OR (“glucagon-like peptide-1 receptor agonist” OR “glucagon-like peptide-1 receptor agonists” OR “GLP-1 receptor agonist” OR “GLP-1 receptor agonists” OR semaglutide OR liraglutide OR exenatide OR dulaglutide OR tirzepatide OR “ROSE-010”).ti,ab,kw.) AND (pain OR nocicep* OR neuropath* OR osteoarthriti* OR headache* OR migraine* OR “idiopathic intracranial hypertension” OR “visceral pain” OR “irritable bowel syndrome” OR fibromyalgia OR opioid*).ti,ab,kw.
Web of Science Core Collection	All indexed years through 15 May 2026	TS=(“glucagon-like peptide-1 receptor agonist” OR “glucagon-like peptide-1 receptor agonists” OR “GLP-1 receptor agonist” OR “GLP-1 receptor agonists” OR semaglutide OR liraglutide OR exenatide OR dulaglutide OR tirzepatide OR “ROSE-010”) AND TS=(pain OR nocicep* OR neuropath* OR osteoarthriti* OR headache* OR migraine* OR “idiopathic intracranial hypertension” OR “visceral pain” OR “irritable bowel syndrome” OR fibromyalgia OR opioid*)

Search terms were adapted to the indexing system and syntax of each database. For mechanistic searches, the exposure block was broadened to include “glucagon-like peptide-1”, GLP-1, GLP-1R, and exendin-4. Additional targeted searches addressed neuroimmune mechanisms (microglia, TRPV1, and interleukin-10), gastrointestinal and periprocedural safety (gastric emptying, aspiration, and sedation), rehabilitation, sarcopenia, lean mass, and body composition. Reference lists and citation links of key reviews and primary studies were also screened to identify additional relevant publications. The asterisk (*) denotes a truncation wildcard used to retrieve variant word endings.
